# Mouse *in Vivo* Neutralization of *Escherichia coli* Shiga Toxin 2 with Monoclonal Antibodies

**DOI:** 10.3390/toxins5101845

**Published:** 2013-10-22

**Authors:** Luisa W. Cheng, Thomas D. Henderson, Stephanie Patfield, Larry H. Stanker, Xiaohua He

**Affiliations:** Western Regional Research Center, U.S. Department of Agriculture, Agricultural Research Service, 800 Buchanan Street, Albany, CA 94710, USA; E-Mails: luisa.cheng@ars.usda.gov (L.W.C.); thomas.henderson@ars.usda.gov (T.D.H.); stephanie.patfield@ars.usda.gov (S.P.); larry.stanker@ars.usda.gov (L.H.S.)

**Keywords:** monoclonal antibodies, neutralization of Shiga toxins, Shiga toxin-producing *E. coli*, toxicokinetics

## Abstract

Shiga toxin-producing *Escherichia coli* (STEC) food contaminations pose serious health concerns, and have been the subject of massive food recalls. STEC has been identified as the major cause of the life-threatening complication of hemolytic uremic syndrome (HUS). Besides supportive care, there currently are no therapeutics available. The use of antibiotics for combating pathogenic *E. coli* is not recommended because they have been shown to stimulate toxin production. Clearing Stx2 from the circulation could potentially lessen disease severity. In this study, we tested the *in vivo* neutralization of Stx2 in mice using monoclonal antibodies (mAbs). We measured the biologic half-life of Stx2 in mice and determined the distribution phase or *t*_1/2_ α to be 3 min and the clearance phase or *t*_1/2_ β to be 40 min. Neutralizing mAbs were capable of clearing Stx2 completely from intoxicated mouse blood within minutes. We also examined the persistence of these mAbs over time and showed that complete protection could be passively conferred to mice 4 weeks before exposure to Stx2. The advent of better diagnositic methods and the availability of a greater arsenal of therapeutic mAbs against Stx2 would greatly enhance treatment outcomes of life threatening *E. coli* infections.

## 1. Introduction

Shiga toxin-producing *Escherichia coli* (STEC) encompass a group of pathogenic *E. coli* that represents a major public health concern worldwide. Infections with STEC occasionally result in severe symptoms of bloody diarrhea and hemolytic-uremic syndrome (HUS) [1,2], which is defined as the triad of hemolytic anemia, thrombocytopenia, and acute kidney injury [3]. Shiga toxins (Stxs) play an important role in the pathogenesis of these disorders. There are two types of Stxs produced by STEC, Stx1 and Stx2 [4]. Both are encoded by *stx* genes on toxin-converting lambdoid temperate bacteriophages [5] and have an AB_5_ structure [6]. The molecular weight of the holotoxin is about 70 kDa, which consists of a single A-subunit of 32 kDa and 5 identical B subunits of 7.7 kDa. The A-subunit is an enzymatically active *N*-glycosidase that inhibits protein synthesis by cleavage of an adenine base from the 28S rRNA at position 4324 of the eukaryotic ribosomal 60S subunit, resulting in cell death [7,8]. The B-pentamer contains multiple receptor binding sites for globotriaosyl ceramide (Gb_3_) [9] or globotetraosyl ceramide (Gb_4_) [10] expressed on mammalian cells. Despite their structural similarities, Stx1 and Stx2 exhibit significant differences in biological activities. Epidemiological and molecular typing studies indicate that STEC strains producing Stx2 have been associated more closely with HUS than STEC strains producing Stx1 [11,12]. 

Currently, no specific protective treatment has been developed for STEC-induced HUS other than supportive therapy. The effect of antibiotics on HUS is still controversial. A study of 259 children infected with STEC indicates that antibiotic use during STEC infection enhances production and release of Stxs, which eventually increases the frequency and severity of HUS [13]. However, there is also evidence showing that some STEC strains do not release Stxs in response to therapeutic concentrations of antibiotics like ciprofloxacin, meropenem, fosfomycin and chloramphenical [14]. Plasma exchange has not been shown to affect the course of the disease [15]. Novel strategies designed for disease prevention include vaccines [16], use of toxin receptor mimics [17], small molecules that block Stx-induced apoptosis [18], and antibodies against Stx [19]. Unfortunately, most of these potential therapeutics have not been tried in humans and none of them have had any impact on the incidence and severity of human cases of STEC-induced HUS. Recently, Stx was observed in the circulation of children with STEC-HUS. Stx could bind to leukocytes for up to 1 week after the diagnosis of STEC-induced diarrhea [20], which indicates the pivotal role of the toxin in the pathogenesis of disease, justifying the use of mAbs against Stx to prevent HUS in patients infected with STEC. Similar to other toxin-induced diseases [21], little endogenous serum antibody is induced against Stxs following STEC infection [22]. Therefore, passive administration of toxin-neutralizing antibodies should be an effective therapy for HUS. A number of Stx-specific mAbs have been developed and tested for their ability to protect animals from Stx-mediated death [23,24,25,26,27,28,29,30,31,32]. However, a detailed toxicokinetic analysis of un-modified Stx2 in the presence or absence of neutralizing antibodies against this toxin in an animal model has not been fully described in the literature. In this study, we tested and validated a newly developed ELISA for the sensitive detection of Stx2 in mouse sera, determined the half-lives of Stx2 in mice and monitored the clearance of Stx2 from the circulatory system by mAbs. We also showed the efficacy of pre- and post-treatment of Stx2 intoxication with neutralizing mAbs. This information will be useful for preclinical evaluation of immunotherapeutic reagents against Stx2 as a means of protecting susceptible patients from developing HUS.

## 2. Results

### 2.1. Detection of Stx2 in Mouse Serum

**Figure 1:**
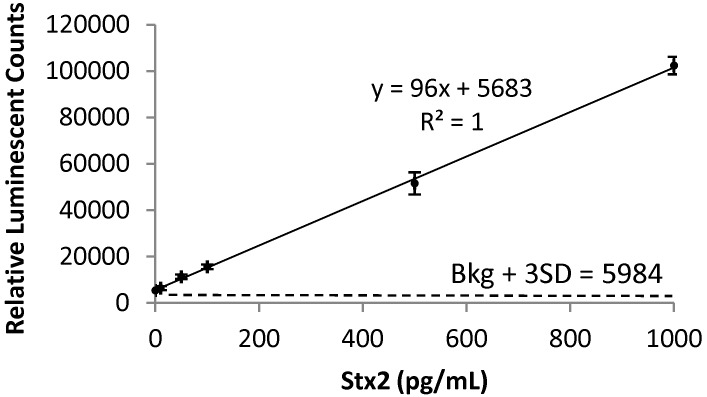
Standard curve of Stx2 spiked in mouse serum. Known standards ranging from 10 to 1,000 pg/mL of Stx2 in control sera (pooled healthy mouse sera) were used to determine the concentration of Stx2 in unknown blood samples. The linear regression of the standard curve has a correlation coefficient (R^2^) of 1. The LOD of 10 pg/mL was determined by the addition of 3 times standard deviation to the mean background signal and is denoted here with a dashed line at 5984 relative luminescent counts.

Currently, diagnosis of STEC infection is determined primarily through isolation of the pathogen from stool culture. STEC strains are distinguished from other *E. coli* strains comprising the normal intestinal flora based on chemical markers, such as the unique sorbitol negative fermentation property of the O157 strain using isolation media [33]. However, this approach is unable to identify non-O157 STEC strains. To determine if a bacterial isolate is a STEC, the best way is to examine the production of Stxs. The availability of an assay that could detect Stxs in the blood system directly may improve the identification of individuals at high risk of HUS during and after a STEC outbreak because of the close association of the Stx with HUS [11,12]. We tried different formats of ELISAs (including direct and indirect ELISA using unlabeled primary and HRP-labeled secondary antibodies, instead of using signal amplification avidin-biotin complex presented in this study) for the detection of Stxs in sera samples and found that our newly developed ELISA [34] was at least 10-fold more sensitive than other formats tested (data not shown). In this study, the LOD determined for Stx2 spiked in mouse sera was 10 pg/mL with a quantification range of 10 to 1,000 pg/mL (Figure 1). 

### 2.2. *In Vivo* Toxicity and Toxicokinetics of Stx2

To determine the toxicity of Stx2 *in vivo*, we administered the toxin intraperitoneally to Swiss Webster mice. The mouse LD_50_ of a commercially available Stx2 was determined as 290 ng/kg or about 6 ng per average sized mouse. Intoxication with Stx2 resulted in weight loss, frequent urination (observed as increased water intake and number of wet cages), and ultimately death. Mice that survived Stx2 challenge recovered weight as well as normal urination behavior.

**Figure 2:**
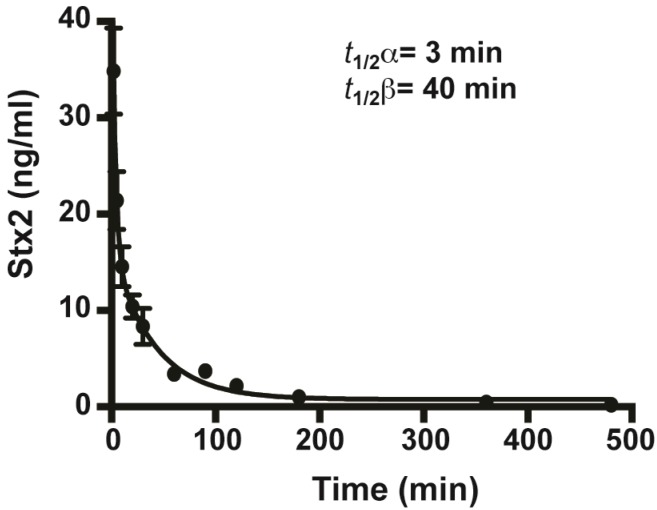
Biologic half-lives of Stx2 in mouse serum. Stx2 was introduced into mice by iv. Sera was taken at 2, 5, 10, 20, 30 min and 1, 1.5, 2, 3, 6 and 8 h after intoxication and the Stx2 concentration was determined based on standard curves plotted in non-linear regression of the second polynomial (Prism 6). The fast distribution phase *t*_1/2_ α and slow clearance phase *t*_1/2_ β were determined using the same program. The mean values for each time point were plotted along with the standard error of the mean (SEM) with *n* ≥ 5.

Little is known thus far about the *in vivo* toxicokinetics of naturally occurring Stx2. Using the sensitive ELISA assay described above, we were able to detect minute amounts of Stx2 in animal sera. Mice treated with 100 ng/mouse of Stx2 via iv were bled and sacrificed over time (2, 5, 10, 20, 30 min and 1, 1.5, 2, 3, 6 and 8 h at *n* ≥ 5 per time point). The concentration of unknown samples was determined by ELISA using a standard curve of known samples diluted in pooled mouse sera. The half-lives, consisting of the distribution phase (*t*_1/2_ α) and the slower clearance phase (*t*_1/2_ β) were determined as 3 min and 40 min, respectively (Figure 2). We observed no statistically significant difference between the concentrations of Stx2 recovered from sera and plasma (data not shown).

### 2.3. Protection of Mice from Stx2 with Monoclonal Antibodies

**Figure 3:**
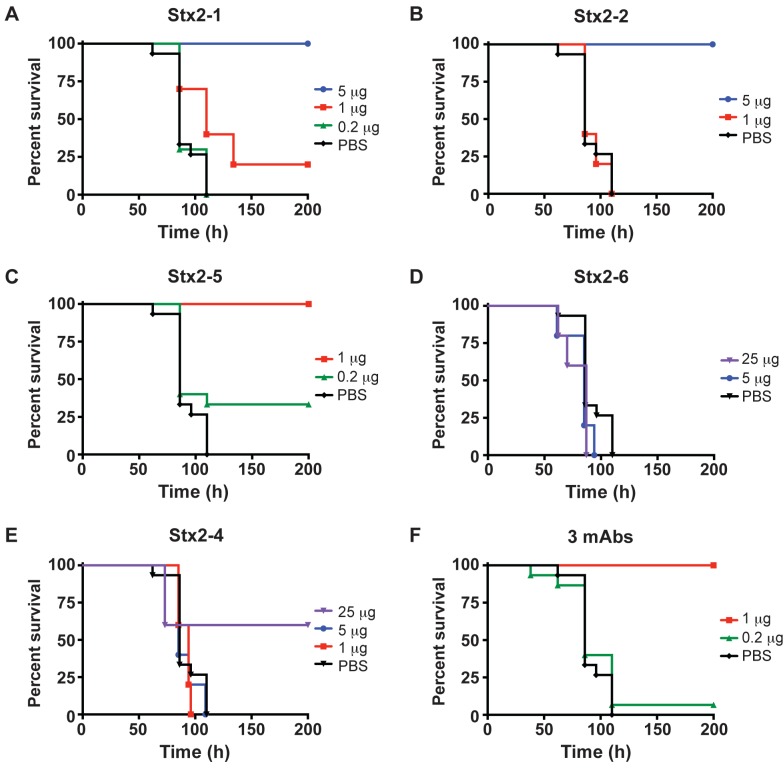
Monoclonal antibody protection of mice from Stx2. Mice (*n* ≥ 10) were treated with different doses of single mAb or with a combination of anti-Stx2 mAbs (**A**. Stx2-1; **B**. Stx2-2; **C**. Stx2-5; **D**. Stx2-6; **E**. Stx2-4 and **F**. 3 mAbs, 1:1:1 of Stx2-1, Stx2-2, and Stx2-5) about 30 min prior to administration with a lethal dose (3 ip mouse LD_50_) of Stx2. The percentage of survival of mice was plotted over time. Control mice were treated with sterile PBS instead of mAb.

In previous studies, we developed five mAbs (Stx2-1, Stx2-2, Stx2-4, Stx2-5, and Stx2-6) for the sensitive detection of Stx2 in immunoassays [34] and (unpublished data). These mAbs were also tested for their ability to neutralize Stx2 activity in Vero cells. Only mAb Stx2-5 showed significant neutralization activity in the cell-based assays [34]. In this study, we tested these mAbs for the *in vivo* neutralization of Stx2. Mice were treated with different doses of a single mAb or a 1:1:1 combination of anti-Stx2 mAbs (Stx2-1, Stx2-2, and Stx2-5) about 30 min prior to ip administration with a lethal dose (3 ip mouse LD_50_) of Stx2. The survival of mice treated with mAbs or sterile PBS were plotted over time (Figure 3). In contrast to the Vero cell toxin neutralization assays, mAbs Stx2-1 and Stx2-2 protected mice well, providing complete protection from death with only 5 µg/mouse of mAbs (Figure 3A and Figure 3B). MAb Stx2-5 provided the highest level of protection, showing full protection at 1 µg/mouse (Figure 3C). MAbs Stx2-4 and Stx2-6 did not provide significant protection from Stx2 even at 25 µg mAb/mouse indicating that the protective effect seen with mAbs Stx2-1, 2 and 5 were not due to the general presence of mAbs (Figure 3D and Figure 3E).

Other studies with antibody protection against botulinum toxin A have shown a substantial additive protective effect of combining two or more mAbs [21,35]. In this study, a combination of the best protective mAbs Stx2-1, Stx2-2 and Stx2-5 conferred complete protection from Stx2 at 1 µg mAb/mouse (Figure 3F). However, this protection was not more significant than using Stx2-5 mAb alone. 

### 2.4. Survival of Mice Treated with mAbs before and after Intoxication with Stx2

**Figure 4:**
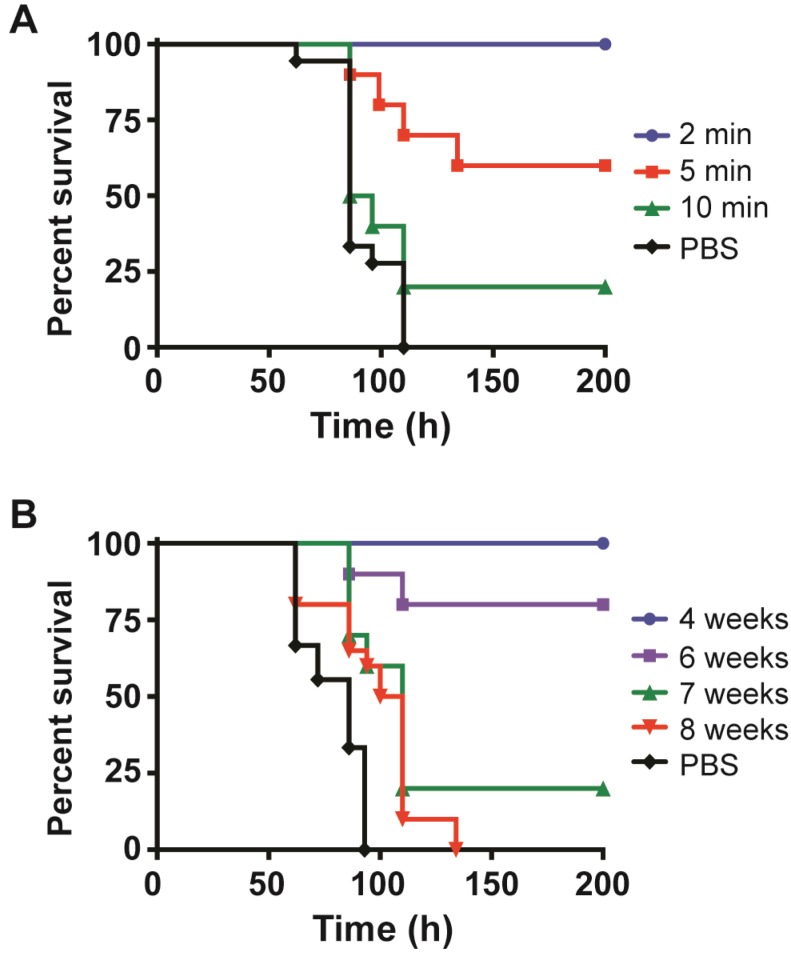
Survival of mice treated with mAbs before and after Stx2 intoxication. A. Mice were treated with a lethal dose of Stx2 followed by treatment with a mAb combination against Stx2 at 2, 5, 10, 20, 30 min and 1 h. B. Mice were treated with the same combination of mAbs against Stx2 at 4, 5, 6, 7, 8 weeks before injection with Stx2.

To elucidate the window of opportunity for mAb protection, we investigated the efficacy of mAbs before and after toxin exposure. Mice treated by iv with a combination of mAbs against Stx2 (3 µg each of mAbs Stx2-1, Stx2-2, and Stx2-5) at 2, 5, 10, 20 and 40 min after injection of Stx2 conferred some degree of protection as shown by the increase of time-to-death (Figure 4A). To make sure a known quantity of Stx2 is in the bloodstream before mAbs were added, the toxin was given by iv. All mice treated with mAbs at 2 min post intoxication (mpi) survived; 60% and 20% of mice survived when treated at 5 and 10 mpi, respectively. All control mice that were treated with PBS instead of mAbs died within 5 days after intoxication (Figure 4A). Mice that received the mAb combination 30 min before Stx-2 treatment, showed no signs of intoxication (data not shown). Significant protection was observed when mAbs were administrated before toxin exposure. Mice were treated with the same combination of mAbs at weeks 3 to 8 before injection with a lethal dose of Stx2 (18 ng/mouse by iv). All mice survived when treated with mAbs at 4 weeks or less before intoxication, while 80% of mice treated with mAbs at 5 and 6 weeks before intoxication survived (Figure 4B and data not shown). Even mice treated with mAbs 7 weeks before intoxication displayed a protective effect as shown by the 20% survival with a slight increase in the median survival from 86 h in the PBS control to 110 h (Figure 4B).

### 2.5. Clearance of Stx2 by Monoclonal Antibodies

**Figure 5:**
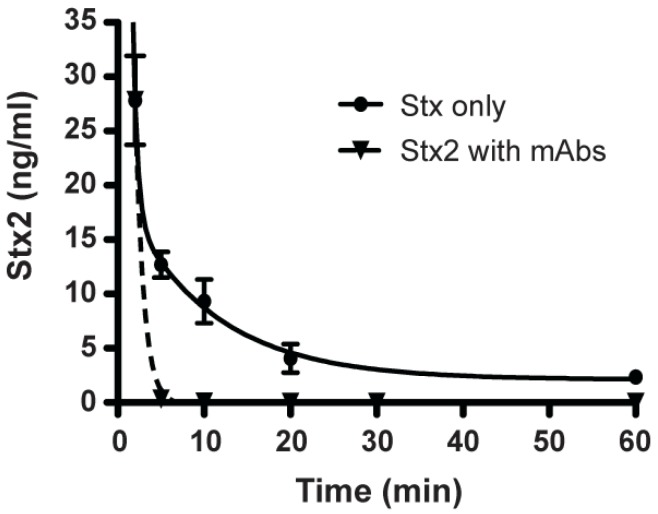
Clearance of Stx2 by monoclonal antibodies. Mice were treated iv with 100 ng of Stx2. They were then treated with (inverted triangles) or without (circles) a combination of mAbs Stx2-1, Stx2-2, and Stx2-5 at 2 min after toxin injection. Sera were obtained at 2, 5, 10, 20, 30 min and 1, and 2 h. MAbs accelerated Stx2 clearance, eliminating toxin from the bloodstream within minutes. The mean values for each time point were plotted along with the standard error of the mean (SEM) with *n* = 3 for no mAb controls and *n* = 6 for mAb treated mice.

To test whether the protection of mice from Stx2 with mAbs is due to the rapid serum clearance of the toxin, we examined the toxicokinetics of Stx2 in the presence or absence of mAbs. Mice were injected with Stx2 by iv, followed by iv introduction of the 3 mAbs combination (Stx2-1, Stx2-2, and Stx2-5) after two min. Sera were obtained at 2, 5, 10, 20, 30 min and 1 h, and the concentration of Stx2 at each time point was determined using the ELISA method described above. Within 3 mpi, the circulating titer of Stx2 went from 13 ± 1.2 ng/mL in the no treatment controls to 0.3 ± 0.05 ng/mL when mAbs were added (Figure 5). At 8 mpi, Stx2 went from 9.3 ± 1.2 ng/mL in nontreated animals to 8 ± 3 pg/mL in mAb-treated animals, suggesting that this combination of mAbs protected mice from Stx2 intoxication by accelerating the clearance of toxin from the bloodstream. 

## 3. Discussion

Stx2 is a major virulence factor of STEC associated with severe HUS. Detection of Stx is the most reliable method for diagnosis of STEC infections. However, due to the lack of sensitive detection methods, there is currently no report in the literature describing the serum Stx levels in humans with STEC infection [3]. In this study, we validated a newly developed ELISA for the sensitive detection of Stx2 in mouse sera (Figure 1). Using this method [34], we were able to detect Stx2 as low as 10 pg/mL. This method could be used in the future for the detection of Stx2 in human sera samples, which may aid in identifying those who might develop the HUS. Previously, the biologic half-life of Stx-2 has been determined with ^125^I labeled Stx-2 [30,36,37]. However, iodinated proteins could be biodehalogenated *in vivo*. Using the highly sensitive ELISA method, we determined the biological half-life of unmodified Stx2 in mice. The distribution phase *t*_1/2_ α of 3 min and the clearance phase *t*_1/2_ β of 40 min of Stx2 suggest that this toxin is cleared rapidly from the bloodstream (Figure 2), possibly through distribution to the kidneys or the central nervous system where most of the Stx2 related damage is observed [3,30,37]. The *t*_1/2_ α of 6 and 4 min observed in rats and mice, respectively, were comparable to those observed in this study. The *t*_1/2_ β of 2.6 h was longer in rats and not reported previously in mice [30,36]. The highly specific mAbs developed for detection assays were tested for the *in vivo* protection of mice from Stx2 in this study. Previous studies have shown that these mAbs recognize different epitopes of Stx2. MAb Stx2-1 binds to the A chain while mAb Stx2-2 and Stx2-5 mainly bind to the B chain [34]. Only mAb Stx2-5 showed significant neutralization activity in the cell-based assays. We show here that all Stx2-1, Stx2-2 and Stx2-5 mAbs can individually protect mice from lethal doses of Stx2 (Figure 3). It has been reported that the ability to neutralize Stx2 *in vitro* does not necessarily correlate with the ability to neutralize Stx2 *in vivo* [38]. This discrepancy may be due to different mechanisms involved in these two systems. In the cell-based assays, the Stx2-specific mAbs accomplish their neutralization activity by blocking the enzymatic activity of the Stx2 A-subunit or by competing for receptor-binding sites on the B-subunit with cell receptors, resulting in reduced toxin entry into cells. With *in vivo* studies, one important mechanism for antibodies circulating in bloodstream is to bind the antigen, and antibody-antigen complexes are then cleared by the Fc receptors in the liver and pulled from the circulation [37,39,40]. Thus, it is not necessary for the antibodies to possess the toxin-neutralizing or the receptor binding site blocking capabilities needed in the cell-based assays. 

It has been observed that the binding of multiple mAbs to a toxin molecule accelerates its clearance and increases neutralization [35,40,41]. Our results indicate that mAb Stx2-5 was able to prevent mice from Stx2 toxicity with as little as 1 μg when administered before intoxication with 3 ip LD_50_ of Stx2 (Figure 3). Using a combination of the most potent mAbs (Stx2-1, Stx2-2 and Stx2-5) did not increase neutralization as is observed in antibody neutralization of other toxins [35,40]. This is likely due to the unique structure of the AB_5_ family of toxins, where the single mAb Stx2-5 could bind to each of the five B-subunits of the holotoxin, improving the neutralization efficacy to about the same level as the addition of different mAbs.

There are seven subtypes of Stx2 (a through g) identified so far, but Stx2a, Stx2c, and Stx2d are the subtypes most closely associated with HUS [42]. These subtypes are very similar to each other at the amino acid sequence level and recognized by any one of our mAbs used as antibody pairs in our ELISAs. Even though Stx2-5 mAb alone neutralized Stx2 well in mice, we opted to use a combination of mAbs in our neutralization assays to increase the potential clearance of other Stx2 serotypes. We predict that the combination of these mAbs will be capable of neutralizing Stx2c and Stx2d besides Stx2a toxin tested in this study. 

Using the combination of 3 mAbs against Stx2, we determined the window of opportunity of clearance of mAbs after systemic intoxication with Stx2. The mAb protection data mirrored that of the Stx2 biologic half-life closely. Mice intoxicated iv with 3 mouse ip lethal dose of Stx2 can be completely rescued if mAbs were administered 2 min after toxin (Figure 4A). Our combination of mAbs cleared Stx2 within minutes of introduction into intoxicated mice (Figure 5). The window of rescue opportunity rapidly closes by 5 min after intoxication (Figure 4A), suggesting that Stx2 in free form would be absorbed into target cells rapidly after entering the circulating system. It was reported that piglets and mice were fully protected against STEC infection when treated with Stx2-specific antibodies 24 hours after bacterial challenge, shortly after the onset of diarrhea [24,43]. These results suggest that Stx2 enters the bloodstream after the onset of initial symptoms of STEC infection. Such antibodies may be also capable of protecting humans at risk of developing HUS if it is administered shortly after the onset of diarrhea but before the onset of HUS. We tested the window for neutralization after intoxication to validate the toxicokinetics determined by our ELISA assays (Figure 2). Antibodies can neutralize toxins in the bloodstream but not toxins that have been absorbed by organs. To increase the efficacy of post-exposure therapy, it may be possible to use some neutralizing antibody components or molecules that are small enough to penetrate intoxicated cells and neutralize toxins that have been absorbed.

We tested how long pre-exposure treatment with a modest dose of mAbs (9 μg/mouse) would substantially protect mice from intoxication and found that 80% mice were protected even when mAbs were given 6 weeks prior to intoxication (Figure 4B). Mouse immunoglobulins have a half-life of about 6–8 days *in vivo* correlating very well with our observed timing of Stx2 protection [44]. Thus, mAbs can persist in the circulation for a long time, clearing any Stx2 produced over time by pathogenic bacteria in intestines. This is useful as a preventative measure where ingested food products are known to be contaminated or a patient has tested positive for pathogenic *E. coli* but has not yet shown severe symptoms. Given the fact that most patients develop HUS within 2 weeks after infection of STEC [45,46], a single effective dose of antibodies will be sufficient to prevent or treat severe HUS complications caused by STEC infection. The use of antibiotics is not currently recommended for combating pathogenic *E. coli* due to the likely induction of Stx production [47]. However, the risk of developing HUS might be reduced if the use of antibiotics is combined with antibody therapy. 

## 4. Experimental Section

### 4.1. Experimental Materials

Stx2 toxin was purchased from List Biological Laboratories, Inc. (Lot #1621A1, Campbell, CA). Endotoxin levels were tested by List Biological Laboratories and found to be acceptable. Toxin was reconstituted as suggested by the manufacturer into a 100 ng/µL stock (in 50 mM Tris, 100 mM NaCl, 0.1% Trehalose), aliquoted and frozen at −80 °C until use. Monoclonal antibodies against Stx2 (Stx2-1, Stx2-2, Stx2-4, Stx2-5) were prepared as described [34]. Stx2-6 was also prepared as mAbs Stx2-1 to Stx2-5 (unpublished results). Briefly, antibodies were purified from ascites fluids and diluted in sterile phosphate buffered saline, pH 7.4 (PBS) into indicated doses. Female Swiss Webster mice of 4–5 weeks of age were purchased from Charles River (Portage, MI) and were fed *ad libitum* and housed in standard conditions. Mouse experiments were performed according to animal-use protocols approved by the Institutional Animal Care and Use Committee of the United States Department of Agriculture, Western Regional Research Center.

### 4.2. Determination of Mean Lethal Dose

Groups of at least 10 randomly selected mice were treated by intraperitoneal (ip) injection with 500 µL per dose of serial dilutions of Stx2 (in a range that spans high lethality to no deaths). Mice were monitored for health or death for up to 14 days post-intoxication. The mean lethal dose (LD_50_) was calculated by the Weil and the Reed and Muench method [48,49]. 

### 4.3. Mouse Protection Assay

Groups of at least 10 mice were treated with 100 µL of indicated doses (25, 5, 1 or 0.2 µg per mouse of individual mAbs or combination of mAbs (1:1:1 ratio of Stx2-1, Stx2-2 and Stx2-5) by tail vein injection (iv) about 30 min before iv administration with a 100 µL lethal dose (3 ip mouse LD_50_ or 18 ng/mouse) of Stx2. Mice were monitored over 14 days. Survival curves (Figure 3) were plotted using Prism 6 (GraphPad Software, Inc. La Jolla, CA). 

### 4.4. ELISA for Stx2

ELISA was performed as described previously [34]. Briefly, black NUNC plates were coated with mAb Stx2-1 (100 µL/well of a 5 µg/mL solution in PBS) and incubated overnight at 4 °C. Plates were then treated with 300 µL of blocking buffer containing 3% bovine serum albumin (BSA) in 0.02 M Tris-buffered saline with 0.9% NaCl, pH 7.4 and 0.05% Tween-20 (TBST) and incubated for 1 hour at 37 °C. Next, plates were washed twice with TBST. After toxin standards and samples (100 µL/well in PBS) were added, the plates were incubated for one hour at 37 °C and then washed six times with TBST. Next, a biotinylated detection antibody (mAb Stx2-2) was added (100 µL/well of a 100 ng/mL solution in blocking buffer). The plates were incubated for 1 hour at 37 °C, washed six times with TBST and then 100 µL/well of 1:20,000 dilution of streptavidin-HRP (Invitrogen, Carlsbad, CA) in blocking buffer was added. The plates were incubated for 1 hour at 37 °C. Finally, the plates were washed six times with TBST and SuperSignal West Pico Chemiluminescent Substrate (Pierce, Rockford, IL) was added. The Stx2 standards used ranged from 10 to 1,000 pg/mL diluted in pooled mouse sera (Figure 1). The data represent the mean ± SD of three replicates from each toxin concentration and was plotted. The unknown values were determined from the linear regression. The limit of detection (LOD) was defined as the lowest toxin concentration at which the average ELISA reading was three standard deviations above the negative control. 

### 4.5. Toxicokinetics of Stx2

The biologic half-lives of Stx2 were determined in the presence or absence of mAbs against Stx2. Mice were treated iv with 100 ng per mouse (100 µL of 1,000 ng/mL stock) of Stx2. Blood from sets of at least 6 mice per time point were taken by submandibular bleeding (2, 5, 10, 20, 30 min and 1, 1.5, 2, 3, 6 and 8 h) into serum or plasma collectors (BD, San Jose, CA). Blood was incubated on ice for at least 1 h, centrifuged for 10 min at 3000 x *g* to separate sera from cellular fractions. Sera were then aliquoted and frozen at −80 °C until use. Sera were also collected from untreated mice for use as controls and pooled mouse sera and buffer were used to dilute Stx2 standards. In mAb clearance, a 100 µL sample of 90 µg/mL mAb combination (9 µg of mAbs per mouse made up of 3 µg ea of Stx2-1, Stx2-2 and Stx2-5) in PBS buffer was administered by iv 2 min after toxin. Blood samples were collected from sets of 6 mice at each time point (2, 5, 10, 20, 30 min and 1, and 2 h) as described above. The concentration of unknown Stx2 was determined from known standards curves by ELISA. The averages at each time point were plotted ± standard error of the mean (SEM), with standard curves plotted in non-linear regression of the second polynomial using the GraphPad Prism 6 program. Averages of Stx2 values at 5 min and 1 h time in sera were compared with those in plasma. We found no statistically significant difference in the sample values between plasma and sera (data not shown). The half-lives were determined by calculating two-phase exponential decay over time using Prism 6.

### 4.6. Treatment of Mice Post-intoxication or Pre-intoxication with Stx2 mAbs

To simulate post-intoxication treatment, mice were treated by iv with 100 µL of 180 ng/mL of Stx2. At different time points after toxin injection (2, 5, 10, 20, 40 min), 100 µL per mouse of a combination of mAbs (9 µg/mouse or 3 µg ea of Stx2-1, Stx2-2 and Stx2-5 mAbs) were administered by iv. To simulate pre-intoxication treatment, mice were treated by iv with 100 µL of the same Stx2 mAb combination at 3, 4, 5, 6, 7, and 8 weeks prior to iv treatment with 100 µL of 180 ng/mL Stx2. Mice were then monitored for at least 14 days post-intoxication.
